# Creating COVID-19 Stigma by Referencing the Novel Coronavirus as the “Chinese virus” on Twitter: Quantitative Analysis of Social Media Data

**DOI:** 10.2196/19301

**Published:** 2020-05-06

**Authors:** Henna Budhwani, Ruoyan Sun

**Affiliations:** 1 Department of Health Care Organization and Policy School of Public Health University of Alabama at Birmingham Birmingham, AL United States

**Keywords:** COVID-19, coronavirus, Twitter, stigma, social media, public health

## Abstract

**Background:**

Stigma is the deleterious, structural force that devalues members of groups that hold undesirable characteristics. Since stigma is created and reinforced by society—through in-person and online social interactions—referencing the novel coronavirus as the “Chinese virus” or “China virus” has the potential to create and perpetuate stigma.

**Objective:**

The aim of this study was to assess if there was an increase in the prevalence and frequency of the phrases “Chinese virus” and “China virus” on Twitter after the March 16, 2020, US presidential reference of this term.

**Methods:**

Using the Sysomos software (Sysomos, Inc), we extracted tweets from the United States using a list of keywords that were derivatives of “Chinese virus.” We compared tweets at the national and state levels posted between March 9 and March 15 (preperiod) with those posted between March 19 and March 25 (postperiod). We used Stata 16 (StataCorp) for quantitative analysis, and Python (Python Software Foundation) to plot a state-level heat map.

**Results:**

A total of 16,535 “Chinese virus” or “China virus” tweets were identified in the preperiod, and 177,327 tweets were identified in the postperiod, illustrating a nearly ten-fold increase at the national level. All 50 states witnessed an increase in the number of tweets exclusively mentioning “Chinese virus” or “China virus” instead of coronavirus disease (COVID-19) or coronavirus. On average, 0.38 tweets referencing “Chinese virus” or “China virus” were posted per 10,000 people at the state level in the preperiod, and 4.08 of these stigmatizing tweets were posted in the postperiod, also indicating a ten-fold increase. The 5 states with the highest number of postperiod “Chinese virus” tweets were Pennsylvania (n=5249), New York (n=11,754), Florida (n=13,070), Texas (n=14,861), and California (n=19,442). Adjusting for population size, the 5 states with the highest prevalence of postperiod “Chinese virus” tweets were Arizona (5.85), New York (6.04), Florida (6.09), Nevada (7.72), and Wyoming (8.76). The 5 states with the largest increase in pre- to postperiod “Chinese virus” tweets were Kansas (n=697/58, 1202%), South Dakota (n=185/15, 1233%), Mississippi (n=749/54, 1387%), New Hampshire (n=582/41, 1420%), and Idaho (n=670/46, 1457%).

**Conclusions:**

The rise in tweets referencing “Chinese virus” or “China virus,” along with the content of these tweets, indicate that knowledge translation may be occurring online and COVID-19 stigma is likely being perpetuated on Twitter.

## Introduction

Stigma is the deleterious, structural force that devalues those who hold undesirable characteristics [1]. Stigma is a social process that occurs between groups; this process can occur in-person and online [2-6]. Regardless of setting, research has consistently found that stigma is associated with negative health outcomes [2,4,6-9]. For example, HIV-related stigma has pushed the HIV-epidemic underground, fueling ongoing transmission [10], and other disease-related stigmas are associated with negative health outcomes ranging from missed clinical visits to suicidal ideation [1,6,9]. There is evidence to show that stigma can become internalized, and internalized stigma can lead to distrust of health professionals, skepticism of public health systems, and an unwillingness to disclose behaviors related to transmission [2,8,9]. Because the coronavirus disease (COVID-19) is infectious, contact tracing is critically important to assessing community spread; thus, it is imperative that individuals trust their public health and health care systems so that they are willing to accept testing and, if diagnosed with COVD-19, report their whereabouts and activities. Therefore, creating and perpetuating stigma related to COVID-19 could be detrimental to public health efforts that require potentially stigmatized individuals to engage with their health systems.

On March 16, 2020, the president of the United States referred to the novel coronavirus as the “Chinese virus” on Twitter. He tweeted “The United States will be powerfully supporting those industries... that are particularly affected by the Chinese Virus...” After this presidential reference, a dialogue emerged examining if the phrase “Chinese virus” was xenophobic and stigmatizing, considering the availability of alternative scientific names such as coronavirus or COVID-19. Since stigma is created and perpetuated by society through social interaction and public commentary (eg, use of the term “Chinese virus” instead of scientific terms on Twitter), and stigma is reinforced by those in power (eg, use of the term “Chinese virus” by the US president), we hypothesized that there would be an increase in the frequency of the phrases “Chinese virus” and “China virus” on Twitter, comparing the prevalence of these phrases before and after the presidential reference.

## Methods

### Twitter

Twitter is an online social media platform where users send and receive short posts (maximum 280 characters) called tweets. Twitter currently has 152 million daily users, who produce about 500 million daily tweets [11].

### Data, Tweets

We downloaded tweets from all 50 US states, using the Sysomos software (Sysomos, Inc). We extracted tweets that mentioned “Chinese virus” or “China virus” but did not contain “COVID-19” or “coronavirus.” The list of keywords referencing the “Chinese virus” are “Chinesevirus,” “Chinese virus,” “Chinavirus,” “China virus,” “#ChineseVirus19,” “#Chinesevirus,” “#ChineseVirusCorona,” and “#Chinavirus.” We excluded tweets containing the keywords “coronavirus,” “corona virus,” “COVID-19,” “COVID19,” “#COVID2019,” and “#corona.” By excluding tweets that contained both “Chinese virus” and “coronavirus,” we collated a sample of tweets that represented the intent of using “Chinese virus” in place of a scientific alternative, likely indicating deliberate stigmatization. We imputed the location of tweets based on Twitter users’ self-reported state of residence. Tweets posted between March 9 and March 15, 2020 (preperiod), were compared with tweets posted between March 19 and March 25, 2020 (postperiod). Original tweets and quote tweets (adding comments to an existing tweet) were included but not retweets (reposting of an existing tweet). Our final sample (N=193,862) contained all tweets posted in the pre- and postperiods by US-based Twitter users that exclusively mentioned a derivative of “Chinese virus.” Data extraction was conducted on April 10, 2020. Ethical approval was provided by the University of Alabama at Birmingham Institutional Review Board (IRB-#300005071).

### Analysis

We used Stata 16 (StataCorp) to analyze our Twitter data and Python software (Python Software Foundation) to plot our state-level gradient heat map.

## Results

**Figure 1:**
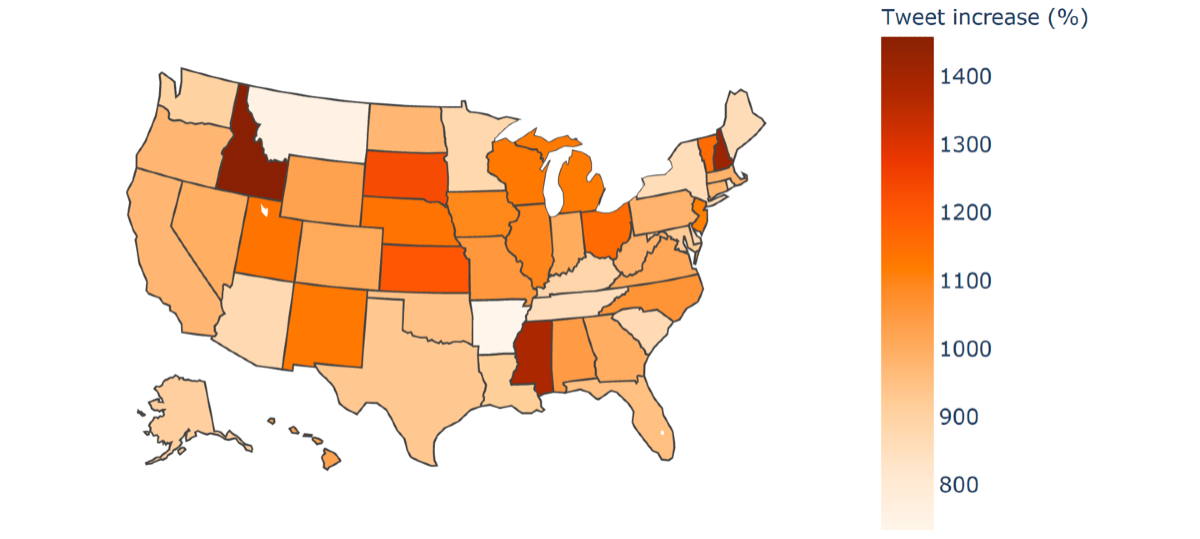
Heat map of increases in tweets referencing “Chinese virus” or “China virus” across the United States.

A total of 16,535 “Chinese virus” or “China virus” tweets were identified in the preperiod, and 177,327 tweets were identified in the postperiod, illustrating a 972.43% (n=160,792/16,535) increase. Comparatively, the number of tweets referencing COVID-19 in the preperiod and postperiod remained steady, at about 4.9 million tweets per period. A total of 13,569 (82.06%) of the preperiod and 145,521 (82.06%) of the postperiod tweets were associated with a Twitter user’s self-reported US state. Figure 1 is a heat map illustrating the state-by-state increases of tweets referencing “Chinese virus” or “China virus.” The darker the shade, the greater the increase. All 50 US states witnessed an increase in the number of tweets exclusively mentioning “Chinese virus” or “China virus” rather than COVID-19 or coronavirus. The 5 US states with the highest number of postperiod “Chinese virus” tweets were Pennsylvania, New York, Florida, Texas, and California. The 5 US states with the largest increase in pre- to postperiod “Chinese virus” tweets were Kansas, South Dakota, Mississippi, New Hampshire, and Idaho.

In Table 1, we present US state-level results of tweets referencing “Chinese virus” or “China virus.” On average, at the state level, 271 such tweets were found in the preperiod and 2910 in the postperiod, indicating a ten-fold increase, similar to what we found at the national level. We also calculated the percentage increase and the prevalence increase. The percentage increase measures the percentage of all COVID-19 related tweets that mentioned “China virus” or “Chinese virus” exclusively. To account for variations in population size, prevalence of “Chinese virus” tweets per 10,000 people for each US state was calculated using the following formula: 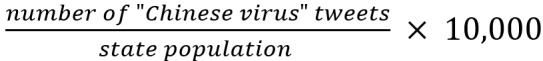. State population sizes were taken from the 2019 US Census Bureau estimates [12]. On average, the state-level percentage increase was 997%, with a minimum of 661% and a maximum of 1447%. Similarly, the prevalence increase mean was 1015%, with a minimum of 734% and a maximum of 1456%. Large variations were found across US states, with the lowest postperiod prevalence of “Chinese virus” or “China virus” in South Dakota and the highest in Wyoming. The 5 US states with the highest prevalence of “Chinese virus” or “China virus” postperiod tweets were Arizona, New York, Florida, Nevada, and Wyoming.

**Table 1:** Tweets referencing the novel coronavirus as “Chinese virus” or “China virus” by state.

States	Preperiod					Postperiod					Change from pre- to postperiod	
	COVID-19 tweets, n	“Chinese virus” tweets, n	Percentage of tweets^a^, (%)	Prevalence of tweets^b^	COVID-19 tweets, n		“Chinese virus” tweets, n	Percentage of tweets^a^, (%)	Prevalence of tweets^b^	Percentage increase^c^ (%)		Prevalence increase^d^ (%)
AL	40,588	153	0.38	0.31	39,434		1749	4.44	3.57	1077		1043
AK	9251	40	0.43	0.55	9597		404	4.21	5.52	874		910
AZ	83,019	438	0.53	0.60	89,127		4256	4.78	5.85	805		872
AR	21,810	109	0.50	0.36	22,741		910	4.00	3.02	701		735
CA	696,645	1806	0.26	0.46	685,596		19,442	2.84	4.92	994		977
CO	84,092	291	0.35	0.51	85,014		3218	3.79	5.59	994		1006
CT	40,304	116	0.29	0.33	40,531		1253	3.09	3.51	974		980
DE	9789	31	0.32	0.32	10,095		304	3.01	3.12	851		881
FL	270,723	1243	0.46	0.58	294,652		13,070	4.44	6.09	866		951
GA	135,543	382	0.28	0.36	136,875		4192	3.06	3.95	987		997
HI	15,261	53	0.35	0.37	18,237		597	3.27	4.22	843		1026
ID	13,810	46	0.33	0.26	14,683		716	4.88	4.01	1364		1457
IL	176,425	410	0.23	0.32	169,849		4918	2.90	3.88	1146		1100
IN	58,767	192	0.33	0.29	57,218		2118	3.70	3.15	1033		1003
IA	27,552	71	0.26	0.23	27,917		847	3.03	2.68	1077		1093
KS	24,678	58	0.24	0.20	24,694		755	0.31	2.59	1201		1202
KY	45,648	179	0.39	0.40	45,841		1765	3.85	3.95	882		886
LA	51,734	151	0.29	0.32	48,623		1535	3.16	3.30	982		917
ME	16,948	54	0.32	0.40	17,762		520	2.93	3.87	819		863
MD	75,527	189	0.25	0.31	76,274		1932	2.53	3.20	912		922
MA	138,665	295	0.21	0.43	137,279		3201	2.33	4.64	996		985
MI	108,514	297	0.27	0.30	103,934		3623	3.49	3.63	1174		1120
MN	63,304	192	0.30	0.34	65,570		1882	2.87	3.34	846		880
MS	19,530	54	0.28	0.18	18,771		803	4.28	2.70	1447		1387
MO	68,869	201	0.29	0.33	71,951		2317	3.22	3.78	1003		1053
MT	9365	61	0.65	0.57	10,503		521	4.96	4.87	662		754
NE	19,791	54	0.27	0.28	18,840		670	3.56	3.46	1203		1141
NV	52,996	217	0.41	0.70	53,730		2377	4.42	7.72	980		995
NH	14,260	41	0.29	0.30	15,096		623	4.13	4.58	1335		1420
NJ	96,806	315	0.33	0.35	100,334		3823	3.81	4.30	1071		1114
NM	18,966	51	0.27	0.24	20,220		627	3.10	2.99	1053		1129
NY	487,901	1225	0.25	0.63	484,515		11,754	2.43	6.04	866		860
NC	110,832	327	0.30	0.31	115,394		3795	3.29	3.62	1015		1061
ND	5649	18	0.32	0.24	6148		193	3.14	2.53	885		972
OH	145,371	366	0.25	0.31	127,421		4613	3.62	3.95	1338		1160
OK	33,480	137	0.41	0.35	33,857		1436	4.24	3.63	937		948
OR	64,817	185	0.29	0.44	65,972		1985	3.01	4.71	954		973
PA	159,712	485	0.30	0.38	161,156		5249	3.26	4.10	973		982
RI	14,234	43	0.30	0.41	14,219		385	2.71	3.63	796		795
SC	43,104	222	0.52	0.43	46,251		2145	4.64	4.17	800		866
SD	6252	15	0.24	0.17	6573		200	3.04	2.26	1168		1233
TN	82,478	361	0.44	0.53	82,050		3431	4.18	5.02	855		850
TX	378,047	1442	0.38	0.50	369,006		14,861	4.03	5.13	956		931
UT	30,422	81	0.27	0.25	28,464		1004	3.53	3.13	1225		1140
VT	8625	18	0.21	0.29	9527		226	2.37	3.62	1037		1156
VA	97,602	301	0.31	0.35	104,176		3351	3.22	3.93	943		1013
WA	123,025	331	0.27	0.43	116,656		3316	2.84	4.35	957		902
WV	15,523	47	0.30	0.26	15,698		509	3.24	2.84	971		983
WI	51,670	130	0.25	0.22	52315		1593	3.05	2.74	1110		1125
WY	6185	45	0.73	0.78	6875		507	7.37	8.76	914		1027
Mean	87,482	271	0.33	0.38	87,545		2910	3.57	4.08	997		1015

^a^Percentage of all COVID-19 related tweets that mentioned “Chinese virus” or “China virus” exclusively.

^b^Prevalence of “Chinese virus” tweets per 10,000 people was calculated using the following formula: 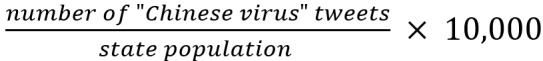.

^c^Percentage of increase was calculated as: 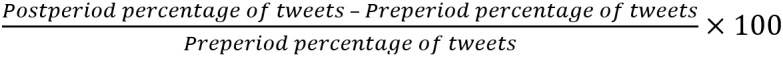.

^d^Prevalence increase was calculated as: 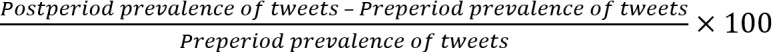.

## Discussion

### Principal Result

We found notable increases in the use of the terms “Chinese virus” and “China virus” on Twitter at both the national and state levels by comparing these tweets (percentage and prevalence) both before and after the March 16, 2020, presidential reference. The following are examples of “Chinese virus” or “China virus” tweets:

- Not parroting MSM's [main stream media’s] narrative. It's the #WuFlu #ChineseCoronaVirus #ChinaVirus”
- “#ChinaVirus #ChinaLiesPeopleDie”

### Limitations

The pandemic is currently underway, so Twitter data—both in quantity (quantitative) and content (qualitative)—are rapidly shifting. We were unable to screen for automatically generated tweets (bots) within this short report [13,14]. Geographic locations associated with Twitter accounts were self-reported; thus, it is possible that some Twitter users may have moved without updating their state location or may have reported a false state location.

### Comparison With Prior Work

There is a growing body of academic literature that leverages Twitter data to assess trends in population health and public sentiment [15-17]. Chew and Eysenbach [18] conducted a seminal examination of knowledge translation using Twitter data during the H1N1 outbreak; they found the proportion of tweets using “H1N1” increased over time compared to the relative use of “swine flu,” suggesting that the media’s choice in terminology (shifting from using the term “swine flu” to “H1N1”) influenced public uptake. In addition, it is relevant that a recent publication by Logie and Turan [19] presented a narrative on how stigma can hurt the COVID-19 public health response. This short report was developed considering the findings from prior studies.

### Future Research

Future research could evaluate and show that stigma mechanisms work online, validate if Twitter and social media data can be informative to epidemic surveillance and health communication, examine the extent that Twitter and social media data is reliable in informing public health efforts and social science research, and explore how Twitter users view COVID-19 and the COVID-19 public health response (eg, testing, linkage to care).

Additionally, although there is a growing body of research using tweets to examine aspects of the novel coronavirus [20-22], to our knowledge, no studies have included a comprehensive set of search terms, which may include phrases such as “ncov,” “covid,” “sars-cov,” and “rona,” in defining their samples. If data extraction is not comprehensive, we run the risk of missing emerging sentiments and terminology, such as referencing the novel coronavirus as the “China virus” or “Chinese virus,” and sociobehavioral outcomes related to these trends.

### Conclusions

The rise in tweets citing “Chinese virus” or “China virus” instead of COVID-19 or the novel coronavirus after the presidential reference on Twitter, along with the content of these tweets, indicate that knowledge translation may be occurring online and COVID-19 stigma is likely being perpetuated on Twitter. Generally speaking, perpetuating COVID-19-related stigma by using the phrase “Chinese virus” could harm public health efforts related to addressing the pandemic, specifically inciting fear and increasing distrust of public health systems by Chinese and Asian Americans. If these stigmatizing terms persist as malicious synonyms for the novel coronavirus, reparative efforts may be required to restore trust by marginalized communities.

## Abbreviations

COVID-19: coronavirus disease
